# Efficacy and safety of auricular point acupressure treatment for gastrointestinal dysfunction after laparoscopic cholecystectomy: study protocol for a randomized controlled trial

**DOI:** 10.1186/s13063-016-1404-3

**Published:** 2016-06-07

**Authors:** Yuhua Tan, Ye Zhao, Tian He, Yueshen Ma, Wang Cai, Yandong Wang

**Affiliations:** Graduate School, Tianjin University of Traditional Chinese Medicine, Nankai District, Tianjin, 300193 China; Department of Clinical Research, Nankai Hospital, Tianjin Academy of Integrative Medicine, No. 6 Changjiang Road, Nankai District, Tianjin, 300100 China; Department of Surgery, Nankai Hospital, Tianjin Academy of Integrative Medicine, Nankai District, Tianjin, 300100 China

**Keywords:** Abdominal pain, Auricular point acupressure, Laparoscopic cholecystectomy, Postoperative gastrointestinal dysfunction, Time to first passage of flatus

## Abstract

**Background:**

Practitioners of traditional Chinese medicine know that auricular point acupressure (APP) using vaccaria seeds on the large intestine point (CO7) has a significant effect on postoperative gastrointestinal dysfunction. A standardized, clinical, research design will transform this clinical experience into scientific evidence, thus providing a basis to promote the wider use of this therapy. We aim to carry out a double-blind, randomized, controlled trial (RCT) to evaluate the efficacy and safety of APP treatment for gastrointestinal dysfunction after laparoscopic cholecystectomy.

**Methods/design:**

This study is a randomized, double-blind, controlled, single-center, clinical, pilot trial. It has been designed according to the Consolidated Standards of Reporting Trials (CONSORT 2010) guidelines as well as the Standards for Reporting Interventions in Controlled Trials of Acupuncture (STRICTA). Study subjects are being selected from among hospitalized patients who have undergone laparoscopic cholecystectomy at the Department of Minimally Invasive Surgery of Tianjin Nankai Hospital. Qualified subjects will be assigned randomly either to the APP group or to the APP sham stimulation group on the basis of random numbers generated using SPSS 19.0. A specifically appointed investigator will be responsible for the randomization. The APP therapy (or sham stimulation) will be performed 6 h after surgery and every 12 h subsequently; six sessions will be conducted, each lasting 3 min. The first evaluation will be performed immediately before the first treatment (6 h after surgery) and, then, every 12 h for seven evaluations. The primary outcome is the time to first passage of flatus after surgery; the secondary outcome measures are abdominal distension, nausea, vomiting, time to first defecation, psychological status, and quality of life.

**Discussion:**

This pilot trial is a standardized, scientific, clinical trial designed to evaluate the efficacy and safety of APP treatment—using vaccaria seeds on CO7—for gastrointestinal dysfunction after laparoscopic cholecystectomy. We aim to provide objective evidence to promote this therapy in clinical practice.

**Trial registration:**

Chinese Clinical Trial Registry, ChiCTR-IPR-15007643. Registered on 14 December 2015.

**Electronic supplementary material:**

The online version of this article (doi:10.1186/s13063-016-1404-3) contains supplementary material, which is available to authorized users.

## Background

Gastrointestinal dysfunction is a common complication of abdominal surgery; its main symptoms include delayed flatus and defecation, abdominal distention, abdominal pain, nausea, and vomiting. Therefore, promoting rapid recovery from gastrointestinal function has become integral to postoperative rehabilitation [1]. Gastric motility typically recovers within 24–48 h after surgery, whereas colonic motility is generally restored within 48–72 h [2]. Thus, it often takes 2–3 days for the patient to recover from gastrointestinal dysfunction. Postoperative gastrointestinal dysfunction is usually treated using alvimopan; however, this drug has adverse effects such as nausea and vomiting [3].

Auricular point acupressure (APP) may be a noninvasive physical treatment for the condition of postoperative gastronintestinal dysfunction [4, 5]. This therapy involves stimulating sensitive points on the auricle using vaccaria seeds; the points correspond to specific organs. In previous studies, we compared subjects’ postoperative time to flatus between an experimental group that received APP using vaccaria seeds on the large intestine point (CO7; 20.19 ± 3.14) and a control group that received APP sham stimulation (30.82 ± 5.58). Preliminary results showed that the APP using vaccaria seeds on CO7 can promote the recovery of gastrointestinal motility after abdominal surgery.

## Methods/design

### Design

**Fig. 1:**
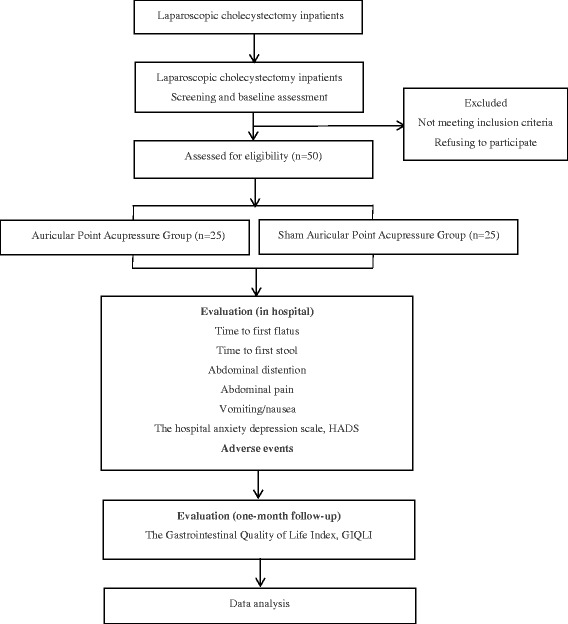
Flow chart of the study

This study is a randomized, double-blind, controlled, single-center, clinical, pilot trial (Fig. 1), which was designed in accordance with the Consolidated Standards of Reporting Trials (CONSORT 2010) guidelines as well as the Standards for Reporting Interventions in Controlled Trials of Acupuncture (STRICTA) [6]. As the Standard Protocol Items: Recommendations for Interventional Trials (SPIRIT) checklist shows in Additional file 1, we are selecting in-patients who have undergone laparoscopic cholecystectomy (LC) between January 2016 and June 2016 at the Department of Minimally Invasive Surgery of Tianjin Nankai Hospital. The APP will be performed by specialist nurses who have more than 5 years of experience and have held intermediate professional titles.

Patients will be randomly assigned either to the APP group or to the APP sham stimulation group on the basis of numbers randomly generated using SPSS 19.0. A specifically appointed investigator will be responsible for the randomization. The APP (or sham stimulation) will be performed 6 h after surgery and every 12 h subsequently; six sessions will be conducted, each lasting 3 min. The first evaluation will be performed immediately before the first APP session (6 h after surgery), and then every 12 h for seven evaluations.

### Study subjects

According to the data from the preliminary study, and given that the primary outcome is the time to the first passage of flatus, the estimated sample size of this study should be 50 cases, with an α value of 0.05, a power of 80 %, and a 15 % dropout rate.

In order to ensure the accuracy of the results, the following inclusion and exclusion criteria have been established.

#### Inclusion criteria

Subjects who are being enrolled in this clinical trial (1) must be age 18 to 65 years; (2) fulfill the classification criteria of the American Society of Anesthesiologists I − II and be scheduled to undergo LC under general anesthesia [7]; (3) have no experience of APP before this study; (4) are not involved in any other clinical trials during the same period; and (5) do not have cognitive impairment, aphasia, or mental disorders, thereby possessing unimpaired communication ability, and can understand and sign the informed consent form.

#### Exclusion criteria

Subjects are excluded if they (1) are pregnant or lactating; (2) have a history of abdominal surgery; (3) have undergone more procedures than LC alone as part of their treatment; (4) have sustained damage to the selected acupressure point or have an allergy to the adhesive; (5) suffer from severe gastrointestinal or chronic pain disorders (such as chronic pelvic pain), and have taken analgesic drugs during the past 48 h—or over a long period of time; (6) have a serious disease of the heart, brain, liver, kidney, or hematopoietic system; or (7) have a history of drug abuse (e.g., morphine), motor-induced or surgery-induced vomiting, obesity (body weight > 80 kg), diabetes, or are bedridden.

#### Elimination criteria

Subjects will be removed from the study if they (1) switch to laparotomy; (2) refuse to continue this treatment after inclusion, regardless of the reason; (3) experience serious complications during treatment (e.g., peritonitis or biliary fistula); (4) are transferred for other specialist treatment, became comatose, or die; (5) cannot attend follow-up visits, for whatever reason; (6) do not meet the inclusion criteria but were included by mistake; or (7) show poor clinical compliance (failure to adhere to the treatment protocol or to provide complete information), which may have affected the efficacy and safety of the treatment.

### Ethical issues

The study was approved by Tianjin Nankai Hospital Institutional Review Board (IRB) on 17 December 2015 (IRB Approval No. 201504901P). After IRB approval had been obtained, this study was registered at an authoritative clinical trial registration platform (the Chinese Clinical Trial Registry) before the start of the study (ID: ChiCTR-IPR-15007643). Informed consent forms were developed in accordance with the requirements of the Declaration of Helsinki. All qualified subjects can freely choose whether or not to participate in this study and will be required to sign the informed consent form before taking part.

### Randomization

Complete randomization will be performed using the random number generator in SPSS 19.0; patients will be randomly assigned to the APP therapy group or the APP sham stimulation group. To reduce selection bias and confounding factors, the randomization will be performed by a single specifically appointed investigator. Each group will contain an equal number of participants. When a qualified subject participates in the trial, the recruiters will obtain the subject’s sequence number from the assigning investigator; the two groups will be assigned to separate wards to prevent communication between them. Thus, bias will be reduced further.

To prevent researcher bias from confounding the results, the randomization will be blinded using the sealed envelope method: the subject recruiters and evaluators will not be informed of the allocation sequence, and the allocation sequence will be placed in sequentially numbered, opaque, sealed envelopes (to ensure that the contents cannot be seen, even under bright light). To prevent the allocation sequence from being disrupted, the subjects’ names and dates of birth will be written on the envelopes, the envelopes will be sealed, and detailed information regarding the subjects will be recorded in a video. The information will be copied onto assignment cards, which are contained in the envelopes, using carbon paper, and a second researcher will watch the video to confirm that the envelopes are still sealed after the subjects’ names are written. A video recorder will be used to record the complete process of opening the envelopes.

### Blinding methods

We will ensure that the evaluators of treatment efficacy are unaware of the group assignments of the subjects by (1) blinding the subject data and (2) using independent statisticians. Moreover, the data will be analyzed by statisticians who are unaware of the group assignment. Thus, operators, evaluators, data administrators, and statisticians will work independently in this study.

### Intervention

**Fig. 2:**
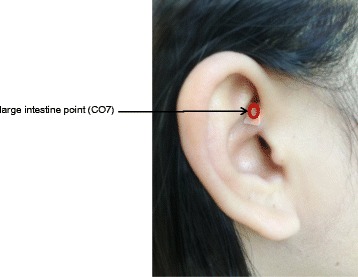
Auricular point acupressure

After surgery, all the included subjects will lie in the supine position without a pillow and fast. The subjects will be given a liquid diet on the day after surgery. The APP therapy (or sham therapy) will be performed 6 h after the operation and every 12 h subsequently, for six treatment sessions, each lasting 3 min. The first evaluation will be performed 6 h after the operation, immediately before the first treatment. The patients will be then evaluated once every 12 h, for seven evaluations. The acupuncture points will be selected on the basis of the Nogier auricular point diagram [8]. Specifically, CO7 will be selected (the anterior 1/3 between the helix crus/part of the helix and the AB line, i.e., the auricular concha zone 7; Fig. 2). Operators will bring the required tools to the subject’s bedside and help the subject assume a comfortable position. The operator will then hold the upper posterior corner of the helix with one hand, and find the sensitive points with an auricular point detector (XS-100A; Guangzhou City Strong Medical Technology Co. Ltd., Guangzhou, China) using the other hand. The area will be disinfected using 75 % alcohol, and the vaccaria seeds will be attached to the auricular points.

### Experimental group

**Fig. 3:**
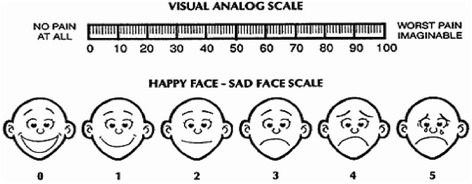
Visual analog scale

Pressure will be applied to both ears alternately without rubbing (to avoid skin damage and infection at the acupuncture point). Optimal pressure will be considered to have been achieved when the subject feels localized tingling pain; the vaccaria seeds will then be fixed to prevent them from coming loose. We use small adhesive tape with spherical vaccaria seeds to adhere to the auricular points, and then, we press the points with digital pressure for 10 s. A 3-s pause occurs between each two pressings. After every intervention, we will remove the tapes. We will use the Visual Analog Scale (VAS; Fig. 3) to evaluate pressure intensity [9]. In accordance with preliminary experiments, the pressure intensity will be controlled at 50 mm. Because the pre-operative fasting time is relatively long, and stimulation of acupuncture points can lead to reduced blood sugar levels, it is necessary to observe whether the subject displays symptoms such as paleness and apathy during the treatment. If such symptoms appear, then the procedure will be ceased immediately, a blood sugar test will be performed, and treatment will be administered as necessary.

### Control group

The subjects in the control group will receive sham APP, i.e., vaccaria-seed tapes adhered to the auricular points with no added pressure. Furthermore, to ensure ethical care and better communication with subjects in the control group, the subjects will be given health education (disease awareness, dietary guidance, exercise, and psychological care) during the treatment.

### Outcome measures and participant timeline

#### Outcome measures

**Fig. 4:**
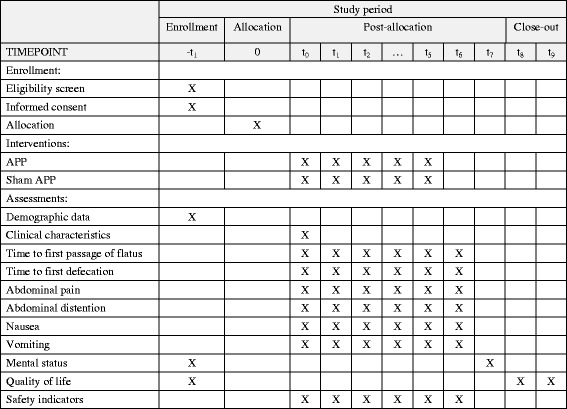
Time schedule of enrollment, interventions, and assessments

1. Baseline data
  - Demographic data will be recorded using a general information questionnaire, which we designed in house. The questionnaire includes questions on age, gender, education, occupation, weight, and height.
  - Clinical characteristics will be recorded using another in-house-designed questionnaire. Specifically, data will be collected on the surgery time, anesthesia time, intraoperative blood loss, intraoperative fluid transfusion volume, and vital signs (temperature, heart rate, respiratory rate, and blood pressure).
2. Primary outcome measure
  The primary outcome is the time to first passage of flatus—we will begin observing and recording when subjects return safely to the ward, and the subjects and their families will be asked to record the time of the first passage of flatus. The interval between the two time points will be then calculated.
3. Secondary outcome measures
  - Time to first defecation: We will observe and record when the subjects return safely to the ward, and the subjects and their families will be asked to record the time of first defecation. The interval between the two time points will then be calculated.
  - Abdominal pain: The subjects’ degree of postoperative pain will be assessed using the VAS scale [10]. The first evaluation will be performed 6 h after surgery, immediately before the first treatment. Evaluations will then be carried out once every 12 h, for seven evaluations. Assistance will be given to subjects and their families in recording this information.
  - Abdominal distention: The Likert scale will be used to assess the subjects’ degree of postoperative abdominal distention [11]. The first evaluation will be performed 6 h after the surgery, immediately before the first treatment. Evaluations will then be carried out once every 12 h, for seven evaluations. Assistance will be given to subjects and their families in recording this data.
  - Nausea: The VAS scale will be used to assess subjects’ degree of postoperative nausea [10]. The first evaluation will be performed 6 h after the surgery, immediately before the first treatment. Evaluations will then be carried out once every 12 h, for seven evaluations. Assistance will be given to subjects and their families in recording this data.
  - Vomiting: The frequency of postoperative vomiting will be recorded. The first evaluation will be performed 6 h after the surgery, immediately before the first treatment. Evaluations will then be carried out once every 12 h, for seven evaluations. Assistance will be given to subjects and their families in recording this data.
  - Mental status: The Hospital Anxiety and Depression Scale (HADS) will be used to assess the subjects’ postoperative mental status [12]. The HADS consists of 14 items: seven assess depression, and the other seven assess anxiety. The scores of the anxiety and depression subscales are classified as follows: 0–7 points indicate no symptoms, 8–10 points indicate suspected symptoms, and 11–21 points indicate confirmed symptoms. The first evaluation will be performed 24 h before surgery, and the second evaluation will be performed on the day of discharge.
  - Quality of life: the Gastrointestinal Quality of Life Index (GIQLI) will be used in this study [13]. The GIQLI aims to measure the quality of life of subjects with digestive diseases and includes five domains: subjective symptoms, physiological functional status, daily life, social activities, and psychological and emotional state. Within these domains, 36 items are assessed, each with a score from 0 to 4 points, for a total possible score of 144 points. The normal population will score 12–125 points. The first evaluation will be performed 24 h before surgery, and the second will be conducted 2 weeks after discharge. A follow-up evaluation will be carried out 1 month after discharge. Higher scores indicate better health status.
4. Safety indicators
  An event can be defined as an adverse event if at least four subjects suffer a symptom—such as local skin allergies, dizziness, and low blood sugar—during the treatment-evaluation period. These events will be statistically analyzed, and treatment safety is evaluated on this basis, in accordance with the research protocol. If serious adverse events occur, they will be immediately reported and managed accordingly.
5. Participant timeline
  For a complete overview of the time schedule of enrollment, interventions, and assessment for participants, see Fig. 4.

### Data collection

To facilitate researchers in observing and recording various indicators, we have organized the content—outcome measures, observation time points, adverse events, and safety evaluations—into a case report form (CRF). Researchers are required to follow the requirements of the CRF as well as fill in the relevant information in a timely and accurate manner.

### Quality control

To ensure the quality of this research, this pilot trial has undergone multiple modifications and revisions by relevant digestive disease specialists, acupuncture experts, professional statisticians, and research methodologists. Strict inclusion and exclusion criteria have been established. To maintain data objectivity, we will ensure that the statisticians and evaluators were blinded. All researchers, and particularly APP operators, have completed our training, thereby ensuring consistent treatment practices and uniformity in the terminology used for communication with the subjects. All operators have a diploma in Chinese Medicine, and they were trained uniformly—before subject recruitment—to pass a test based on the operational protocol. The drugs and materials used in this research have been purchased uniformly; thus, we can ensure that our conclusions will be reliable. The CRF is filled in strictly and in accordance with the CRF instructions; in addition, detailed data regarding subject observations are being recorded. We require the researchers to be conscientious and thorough during data entry, and a corresponding evaluation report is attached. Original data cannot be altered; if modifications are made, these will be explained in detail and accompanied by the signature of the staff member who made the change.

This study involves a two-level quality inspection system. Level 1 involves a quality control inspection. Quality inspectors will be appointed by the study leader; they will design a quality checklist and monitor the records of all original data, data reports, and adverse events in accordance with that checklist. Level 2 involves quality supervision. The quality supervisor will be appointed directly by the study leader. The supervisor will (1) monitor the research operators’ command of the research design and process; (2) confirm the authenticity, accuracy, and integrity of all research data records, reports, and CRF; and (3) ensure consistency in the original data.

### Sample size estimation and statistical analysis

#### Sample size estimation

This study consists of two groups; the sample size-estimation formula is therefore based on a two-group, parallel, controlled design:

$$ n=\frac{{\left({\mathrm{Z}}_{1-a/2}+{\mathrm{Z}}_{1-\beta}\right)}^2\times \left({\sigma}_1^2+{\sigma}_2^2\right)}{\delta^2}, $$

where *n* represents the sample size in each group, σ represents the standard deviation, and *δ* represents the between-group difference with clinical significance. At an α value of 0.05, a power of 80 %, and given a two-sided test, we obtained Z scores of Z_1 − *a*/2_ = 1.96 and Z_1 − *β*_ = 0.84 from the Z score table. Eight subjects recruited from November 2015 to December 2015 were included in the pilot trial, and the primary outcome measure was the time to first flatus: *S*_1_ = 3.14 and *S*_2_ = 5.58. A decrease of 4 h in the time to first flatus was set as our clinical expectation based on preliminary tests and clinical experience. The above data were used to estimate the value of the overall parameter to be inserted into the above formula, and an n-value of 20.09 was obtained. Therefore, the rounded sample size in each group was 21; assuming a 15 % dropout rate, the final sample sizes in the experimental and control groups would be 25 cases, yielding the required 50 cases.

#### Statistical analysis

1. Content of statistical analysis
  - Sample distribution: The absolute number of dropouts as well as the rate will be described for each data set. These data will be analyzed, and the reasons for each termination are listed in detail.
  - Balance comparison: The subjects’ general information will be compared to assess the comparability of the two groups.
  - Efficacy analysis: The subjects’ time to first passage of flatus will be used as the primary outcome measure to evaluate the efficacy and safety of APP in treating gastrointestinal dysfunction after laparoscopic cholecystectomy. The means and standard deviations will be calculated to describe the outcome measure, and the experimental and control groups will be statistically compared using two independent-sample *t* tests. The subjects’ time to first defecation, abdominal pain, abdominal distention, nausea, vomiting, mental status, and quality of life—i.e., the seven secondary outcome measures—will be used to evaluate the efficacy of the APP. Six of these—namely, the time to first defecation, abdominal pain, abdominal distention, nausea, vomiting, and quality of life—will be described using mean ± standard deviation. The scores for mental status will be recorded and categorized in terms of the classification criteria. The statistical analysis of time to first defecation is the same as that of time to first passage of flatus; abdominal pain, abdominal distention, nausea, vomiting, and quality of life will be statistically compared between groups using Repeated Measures Analysis of Variance (RMANOVA). However, before the RMANOVA is performed, a sphericity test will be used to determine whether the data are suitable for analysis using RMANOVA. If the data do not meet the conditions of sphericity, the correction factor *ε* will be used to correct the degree of freedom before the RMANOVA is performed. The categorical measure of mental status will be analyzed using either the rank-sum test or Spearman’s rank correlation.
  - Safety analysis: In accordance with the above definitions of adverse events, we compiled a list describing, grading, and explaining the adverse events in detail. We will ascertain descriptive statistics of the absolute number—as well as the incidence—of each adverse event in both groups. When making comparisons, we will use the *χ*^2^ test or Fisher’s exact test for statistical analysis.
2. Methods of statistical analysis
  Data will be analyzed in accordance with the principle of intention-to-treat (ITT). That is, we will include all subjects who were treated at least once in the trial. Efficacy and safety measures will be analyzed in accordance with “per-protocol” (PP), whereby we will include all subjects who completed the entire trial and followed the protocol requirements. Quantitative variables in the trial data will be expressed as $\overline{\mathrm{X}}\pm \mathrm{S}\mathrm{D}$ for statistical description, whereas qualitative variables will be recorded as the number of cases in each category. If the quantitative variables constitute repeated measures data, then RMANOVA will be used for comparison, and the sphericity test will be used to determine the suitability of, as well as to correct, the data. Qualitative variables will be analyzed using either the rank-sum test or Spearman’s rank correlation; count data for incidence will be compared using the *χ*^2^ test or Fisher’s exact test. All statistical tests are two-sided, and differences with *P* values < 0.05 will be considered statistically significant. Statistical analysis will be performed using SPSS 19.0 statistical software for calculations.

## Discussion

Anesthesia, carbon dioxide pneumoperitoneum, surgical trauma, inflammation, postoperative bed rest, and other factors can cause postoperative gastrointestinal dysfunction in LC patients. Such adverse effects can lead to prolonged hospitalization, prevent patients from recovering functionally, and increase the medical burden.

Currently, postoperative gastrointestinal dysfunction is treated using postoperative fasting, gastrointestinal decompression, early ambulation, enema, chewing gum, gastroprokinetic drugs, and early enteral nutrition [14–16]. In this regard, APP using vaccaria seeds has clear benefits for patients with diseases of the digestive system [17, 18]. This study is the first standardized, scientific, clinical trial to evaluate the efficacy and safety of APP treatment using vaccaria seeds for postoperative gastrointestinal dysfunction. The study was prompted by long-term clinical experience and will use the time to first passage of flatus as the primary outcome measure. We wish to provide a scientific basis upon which to promote this therapy.

A systematic review [1] showed that the time to first passage of flatus has important clinical significance in terms of recovering gastrointestinal motility, and the same parameter is closely related to the recovery of the postoperative gastrointestinal dysfunction. For this reason, most clinical trials addressing the treatment of postoperative gastrointestinal dysfunction, such as that by Cho in 2015 [19] and that by Kim in the same year [20], use the time to first passage of flatus as a primary outcome. In this way, they can evaluate the recovery of gastrointestinal function. Hence, we also have set this measure as the primary outcome in our trial. During the perioperative period, subjects may exhibit psychological changes such as anxiety and depression [21]; thus, the subjects’ anxiety and depression will be used as secondary indicators of recovery. Furthermore, subjects’ quality of life within a month of discharge can also indicate the longer-term effects of this therapy.

### Trial status

The recruitment of this trial is currently ongoing.

## Abbreviations

APP, auricular point acupressure; CRF, case report form; GIQLI, Gastrointestinal Quality of Life Index; HADS, Hospital Anxiety and Depression Scale; LC, laparoscopic cholecystectomy; VAS, visual analog scale

## Additional file

Additional file 1: SPIRIT checklist. (DOC 124 kb)

## References

[1] Vather R, Trivedi S, Bissett I (2013). Defining postoperative ileus: results of a systematic review and global survey. J Gastrointest Surg.

[2] Story SK, Chamberlain RS (2009). A comprehensive review of evidence-based strategies to prevent and treat postoperative ileus. Dig Surg.

[3] Marderstein EL, Delaney CP (2008). Management of postoperative ileus: focus on alvimopan. Ther Clin Risk Manag.

[4] Tan JY, Molassiotis A, Wang T, Suen LK (2014). Adverse events of auricular therapy: a systematic review. Evid Based Complement Alternat Med.

[5] Yeh CH, Morone NE, Chien LC, Cao Y, Lu H, Shen J (2014). Auricular point acupressure to manage chronic low back pain in older adults: a randomized controlled pilot study. Evid Based Complement Alternat Med.

[6] MacPherson H, White A, Cummings M, Jobst K, Rose K, Niemtzow R (2001). Standards for reporting interventions in controlled trials of acupuncture: the STRICTA recommendations. Complement Ther Med.

[7] Bakri MH, Ismail EA, Ibrahim A (2015). Comparison of dexmedetomidine and dexamethasone for prevention of postoperative nausea and vomiting after laparoscopic cholecystectomy. Korean J Anesthesiol.

[8] Santoro A, Nori SL, Lorusso L, Secondulfo C, Monda M, Viggiano A (2015). Auricular acupressure can modulate pain threshold. Evid Based Complement Alternat Med.

[9] Kersten P, White PJ, Tennant A (2014). Is the pain visual analogue scale linear and responsive to change? An exploration using Rasch analysis. PLoS One.

[10] Adib-Hajbaghery M, Etri M, Hosseainian M, Mousavi MS (2013). Pressure to the p6 acupoint and post-appendectomy pain, nausea, and vomiting: a randomized clinical trial. J Caring Sci.

[11] Liu MY, Wang CW, Wu ZP, Li N (2014). Electroacupuncture for the prevention of postoperative gastrointestinal dysfunction in patients undergoing vascular surgery under general anesthesia: study protocol for a prospective practical randomized controlled trial. J Integr Med.

[12] Bjelland I, Dahl AA, Haug TT, Neckelmann D (2002). The validity of the Hospital Anxiety and Depression Scale. An updated literature review. J Psychosom Res.

[13] Yeung SM, Shiu AT, Martin CR, Chu KM (2006). Translation and validation of the Chinese version of the Gastrointestinal Quality of Life Index in patients with gastric tumor. J Psychosom Res.

[14] Short V, Herbert G, Perry R, Atkinson C, Ness AR, Penfold C (2015). Chewing gum for postoperative recovery of gastrointestinal function. Cochrane Database Syst Rev.

[15] Nguyen DL, Maithel S, Nguyen ET, Bechtold ML (2015). Does alvimopan enhance return of bowel function in laparoscopic gastrointestinal surgery? A meta-analysis. Ann Gastroenterol.

[16] Correia MI, da Silva RG (2004). The impact of early nutrition on metabolic response and postoperative ileus. Gastroenterology.

[17] Yeh CH, Chien LC, Chiang YC, Lin SW, Huang CK, Ren D (2012). Reduction in nausea and vomiting in children undergoing cancer chemotherapy by either appropriate or sham auricular acupuncture points with standard care. J Altern Complement Med.

[18] Li MK, Lee TF, Suen KP (2014). Complementary effects of auricular acupressure in relieving constipation symptoms and promoting disease-specific health-related quality of life: a randomized placebo-controlled trial. Complement Ther Med.

[19] Cho JS, Kim HI, Lee KY, An JY, Bai SJ, Cho JY (2015). Effect of intraoperative dexmedetomidine infusion on postoperative bowel movements in patients undergoing laparoscopic gastrectomy: a prospective, randomized, placebo-controlled study. Medicine (Baltimore).

[20] Kim G (2015). Electroacupuncture for postoperative pain and gastrointestinal motility after laparoscopic appendectomy (AcuLap): study protocol for a randomized controlled trial. Trials.

[21] Ay AA, Ulucanlar H, Ay A, Ozden M (2014). Risk factors for perioperative anxiety in laparoscopic surgery. JSLS.

